# Nanostructure of Poly(Acrylic Acid) Adsorption Layer on the Surface of Activated Carbon Obtained from Residue After Supercritical Extraction of Hops

**DOI:** 10.1186/s11671-016-1772-3

**Published:** 2017-01-03

**Authors:** M. Wiśniewska, A. Nosal-Wiercińska, I. Ostolska, D. Sternik, P. Nowicki, R. Pietrzak, A. Bazan-Wozniak, O. Goncharuk

**Affiliations:** 1 Department of Radiochemistry and Colloids Chemistry, Faculty of Chemistry, Maria Curie-Sklodowska University, M.Curie-Sklodowska Sq. 3, 20-031 Lublin, Poland; 2 Department of Analytical Chemistry and Instrumental Analysis, Faculty of Chemistry, Maria Curie-Sklodowska University, M. Curie-Sklodowska Sq. 3, 20-031 Lublin, Poland; 3 Department of Physicochemistry of Solid Surface, Faculty of Chemistry, Maria Curie-Sklodowska University, M. Curie-Sklodowska Sq. 3, 20-031 Lublin, Poland; 4 Laboratory of Applied Chemistry, Faculty of Chemistry, Adam Mickiewicz University in Poznań, Umultowska Street 89b, 61-614 Poznań, Poland; 5 Chuiko Institute of Surface Chemistry, National Academy of Science of Ukraine, 17 General Naumov Street, Kiev, 03164 Ukraine

**Keywords:** Mesoporous activated carbon, Poly(acrylic acid) adsorption, Nanostructure of adsorption layer, Physical activation, Electrokinetic properties

## Abstract

The nanostructure of poly(acrylic acid) (PAA) adsorption layer on the surface of mesoporous-activated carbon HPA obtained by physical activation of residue after supercritical extraction of hops was characterized. This characterization has been done based on the analysis of determination of adsorbed polymer amount, surface charge density, and zeta potential of solid particles (without and in the PAA presence). The SEM, thermogravimetric, FTIR, and MS techniques have allowed one to examine the solid surface morphology and specify different kinds of HPA surface groups. The effects of solution pH, as well as polymer molecular weight and concentration, were studied. The obtained results indicated that the highest adsorption on the activated carbon surface was exhibited by PAA with lower molecular weight (i.e., 2000 Da) at pH 3. Under such conditions, polymeric adsorption layer is composed of nanosized PAA coils (slightly negatively charged) which are densely packed on the positive surface of HPA. Additionally, the adsorption of polymeric macromolecules into solid pores is possible.

## Background

Specific structure of polymeric adsorption layer formed on the solid-liquid interface determines the surface properties of colloidal suspension. This is very important for stability of highly dispersed system occurring commonly in many areas of human activity (i.e., environmental, agricultural, and industrial applications) [1–8]. The possibility of obtaining a desirable structure of polymeric layer on the solid surface results from the fact that macromolecules can assume a great number of different conformations. The unique conformation of polymeric chain is a consequence of rotation of the atoms or atom groups around a single bond. It defines the behavior of the polymer in a solution and results from the interactions of macromolecules with solvent molecules.

The conformation of polymeric chains (especially those classified as ionic polymers) can be influenced by many factors, among which the most essential are as follows: pH and ionic strength of solution; temperature; type, molecular weight, concentration and polydispersity of a polymer; and type, purity, and surface properties of a solid. Changing and controlling of one or more parameters, one can obtain the solid suspension characterized by required stability for specified practical application.

Both non-ionic (i.e., poly(ethylene glycol), polyvinylpyrrolidone, poly(acrylic acid)—its functional groups do not undergo dissociation in aqueous solutions) and ionic (i.e., poly(acrylic acid), polyacrylamide, polyamino acids, proteins—their macromolecules contain ionized groups) polymers are used in basic research in the system containing mineral oxides [9–13]. These were as follows: silicon(IV) oxide, zirconium(IV) oxide, titanium(IV) oxide, manganese(IV) oxide, aluminum(III) oxide, chromium(III) oxide, and iron(III) oxide. The natural and synthetic zeolites are also extensively studied [14–16]. Such systems have a great variety of possible applications—in cosmetics, pharmaceutics, paint production, and medicine as components of implants and drug carriers, as well as water treatment technologies and mineral processing [16–21].

Activated carbons are a class of adsorbents which are important for many practical applications. They have a typically highly developed surface area whose value can go up to 1500 m^2^/g. The shape and size of pores are varied. They may be in the form of open channels on both sides, inkpot shaped, V-shaped, and slots having parallel or non-parallel walls. Taking into account the size of the pores, they were divided into three types: microporous (below 2 nm), mesoporous (2–50 nm), and macroporous (above 50 nm) carbons. The most commonly used raw material for the preparation of activated carbon is coal [22]. Other raw materials used for mass production are as follows: coconut husks and wood. Currently, waste products, such as fruit stones or shells of nuts, are becoming increasingly important [23].

Activated carbons are widely used for removal of industrial waste gases through the adsorption of SO_2_, SO_3_, H_2_S, CS_2_, NH_3_, NO_x_, and other toxic compounds. Purification of wastewaters from aliphatic and aromatic hydrocarbons, phenols and their derivatives, pesticides, detergents, heavy metals, bacteria, viruses, dyes, and low-molecular weight organic compounds is also an important area of activated carbon application [24, 25].

The usage of activated carbon as regards polymeric substance adsorption is extremely rarely reported in the scientific literature. For this reason, the aim of this paper is the determination of adsorption properties of activated carbon (HPA) obtained from the residue after supercritical extraction of hops in the process of physical activation as regards low-molecular weight poly(acrylic acid).

Due to the fact that poly(acrylic acid) exhibits excellent solubility in water, is non-toxic, and is biocompatible with human muscle tissue, it finds a wide application in food processing, medicine, and many branches of industry (cosmetics, pharmaceuticals, paints, pigments, and paper production) [1, 3]. PAA forms pH-sensitive and temperature-sensitive hydrogels with various polymers (i.e., poly(vinyl alcohol), polyvinylpirrolidone, chitosan, cellulose). They find mainly usage in controlled drug delivery systems [26, 27]. Other directions of PAA applications include wastewater purification, mineral processing, metal ion recovery, and improvement of soil quality in agriculture [5, 7, 8].

The applied activated carbon is a low-cost adsorbent prepared from plant waste material, and thus, it is competitive to other—much more expensive—adsorbents. Additionally, studies concerning determination of nanostructure of polymeric adsorption layer on the surface of activated carbon can be helpful in its future application as far as macromolecular compound binding is concerned.

## Methods

### Preparation and Characterization of Activated Carbon

The starting material was the residue after supercritical extraction of hops (H) powder with the size 0.10–0.75 mm, and the moisture content in the air-dry state is 5.6%. The initial material was first subjected to the pyrolysis (P) process at 500 °C. It was carried out in a quartz tubular reactor heated by horizontal furnace under a stream of nitrogen with a flow rate of 170 mL/min. At the final temperature, the sample was kept for 1 h and then, it was cooled in inert atmosphere. Then, the char was subjected to physical activation (A) at a temperature of 800 °C under a stream of carbon dioxide (rate flow 250 cm^3^/min), for 1 h. The adsorbent was denoted as HPA.

To remove the mineral matter deposited on the adsorbent surface, activated carbon was washed with doubly distilled water to achieve the supernatant conductivity about 2 μS/cm (conductivity meter CDM 83, Radiometer).

The solid BET surface area, pore volume, and average pore diameter were determined by the use of nitrogen adsorption/desorption method (Accelerated Surface Area and Porosimetry ASAP 2405 Analyzer, Micrometritics Inc., USA).

The SEM images of activated carbon were obtained using the high-resolution scanning electron microscope Quanta 3D FEG (FEI, Field Electron and Ion Co.).

Thermal analysis of the CSDA sample was made on a Simultaneous Thermal Analyzer STA 449 Jupiter F1 (Netzsch, Germany) under the following operational conditions: heating rate of 10 °C/min, nitrogen stream of flow rate 50 cm^3^/min, temperature range of 30–950 °C, sample weight ~18 mg, and sensor thermocouple type S TG-DSC. The gaseous products emitted during the decomposition of materials were analyzed by means of the FTIR spectrometer (Brucker, Germany) and by Quadrupole Mass Spectrometer QMS 403C (Aeölos, Germany) coupled on-line to the STA instrument. The QMS data were gathered in the range from 10 to 300 amu. The FTIR spectra were recorded in the spectral range of 600–4000 cm^−1^ with 16 scans per spectrum at a resolution of 4 cm^−1^.

The acid-base surface properties were evaluated according to the Boehm method [28]. The volumetric (0.1 mol/dm^3^) standard HCl and NaOH (Avantor Performance Materials Poland S.A.) were used as the titrants.

**Fig. 1:**
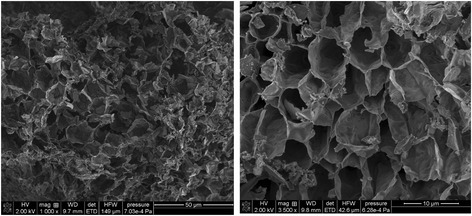
SEM image of activated carbon

The physicochemical characteristics of activated carbon can be found in Table 1. Additionally, the SEM image of activated carbon particles is presented in Fig. 1.

**Table 1:** Physicochemical characteristics of HPA activated carbon

Parameter	Value
BET surface area [m^2^/g]	1169
Total pore volume [cm^3^/g]	0.66
Micropore volume [cm^3^/g]	0.29
Average pore diameter [nm]	2.26
pH	5.5
Acidic group content [mmol/g]	0.47
Basic group content [mmol/g]	1.57

### Characteristics of Polymer

Poly(acrylic acid)—PAA (Fluka), with the two weights of average molecular weight $\left({\overline{\mathbf{M}}}_{\mathbf{w}}\right)$: 2000 Da and 240,000 Da, was used in the study. The applied polymer has weak anionic character—it contains carboxyl groups (p*K*a = 4.5) [29]. Knowing the p*Ka* value, one can determine the degree of carboxyl group ionization under different pH conditions, using the following equation:

1 $$ \alpha \left[\%\right]=\frac{100}{1+{10}^{\left(\mathrm{p}K\mathrm{a}-\mathrm{p}\mathrm{H}\right)}} $$

For p*K*a value determination, the PAA solution (100 ppm) in the supporting electrolyte (NaCl, 0.01 mole/dm^3^) was titrated using NaOH solution (0.1 mol/dm^3^). The obtained titration curve, presenting the dependence of solution pH versus the volume of added base (*V*_*b*_), enables specification of NaOH volume corresponding to the end point of titration (*V*_ep_). The p*K*a value was determined as pH value for which *V*_*b*_ = 1/2*V*_ep_.

The dissociation of PAA functional groups at pH 3 is minimal (dissociation degree (*α*) equaled 0.03), whereas at pH 4.5, numbers of ionized and neutral carboxyl groups are the same (degree of dissociation reaches the value 0.5). The total ionization of polymeric chains occurs at pH 6 and 9 (*α* = 0.97 and *α* = 0.99, respectively) [26].

### Adsorption Measurements

Adsorption measurements were performed at 25 °C in the NaCl solution (concentration of 0.01 mol/dm^3^) at three solution pH values (3, 6, and 9 ± 0.1). First, six series (three for each PAA sample differing in polymer molecular weight) of poly(acrylic acid) solutions with the concentrations ranging from 20 to 500 ppm were prepared. Then, 0.1 g of activated carbon was introduced into each polymer solution. The appropriate pH values of the examined suspensions were adjusted using a pH-meter (Beckman Instruments). The adsorption process was carried out under the conditions of continuous shaking through 24 hours. Next, the solids were centrifuged using a microcentrifuge (MPW Med. Instruments) and the supernatant was collected for further analysis.

The amount of adsorbed PAA was obtained from the difference between the polymer concentration in the solution before and after the adsorption process (using calibration curves). The reaction of poly(acrylic acid) with hyamine proposed by Crummet and Hummel [30] was used. The obtained turbidity was measured at the wavelength 500 nm after 15 min from the initiation of PAA reaction with hyamine, using the UV-VIS spectrophotometer Cary 100 (Varian).

### Electrokinetic Measurements

Potentiometric titration enables determination of the solid surface charge density as a function of solution pH. To obtain a supporting electrolyte curve (as dependence of the changes of pH versus the volume of the added base), 50 cm^3^ of sodium chloride solution (0.01 mol/dm^3^) was placed in the thermostated vessel and titrated with NaOH solution (0.1 mol/dm^3^) [31]. Then, the suspension curve was obtained in an analogous way using 0.035 g of activated carbon. Next, the potentiometric titrations were performed in the HPA–NaCl–PAA systems at the polymer concentrations of 100 and 500 ppm.

The solid surface charge density was determined at 25 °C by the use of a set consisting of thermostated Teflon vessel, glass and calomel electrodes (Beckman Instruments), pH-meter PHM 240 (Radiometer), laboratory stirrer, thermostat RE 204 (Lauda), automatic microburette Dosimat 765 (Metrohm), and a computer with the special software “titr_v3” authored by Prof. W. Janusz.

The activated carbon suspensions (without and with PAA) for the zeta potential measurements were prepared by adding 0.01 g of the solid to 100 cm^3^ of the supporting electrolyte or polymer solution (with the concentration of 100 or 500 ppm). The suspensions were sonicated for 5 min applying an ultrasonicator XL 2020 (Misonix). Then, the solution was divided into seven parts and poured into the Erlenmayer flasks, following that the appropriate pH (3, 4, 5, 6, 7, 8, 9 ± 0.1) was adjusted. The ζ potential of systems prepared in this way was measured using Zetasizer Nano ZS (Malvern Instruments) at 25 °C. Zeta potential value was calculated on the basis of electrophoretic mobility of solid particles in the liquid medium using the Smoluchowski equation [32].

For electrophoretic mobility determination, the technique of laser Doppler electrophoresis was used. The movement of charged particles in an electric field is characterized on the basis of Doppler effect. The frequency shift of light scattered from a moving particle was determined. For this purpose, a laser beam emitted from a single source is divided into two mutually coherent beams. One of them passed through particle dispersion (scattering beam), whereas the latter one (reference beam) routed around the measuring cell. These two beams were superimposed, and their interference occurs. It induced a modulated signal with a frequency equal to the difference between frequencies of the reference and scattering beams. A big difference is equivalent to high electrophoretic mobility.

## Results and Discussion

### Properties of Activated Carbon

**Fig. 2:**
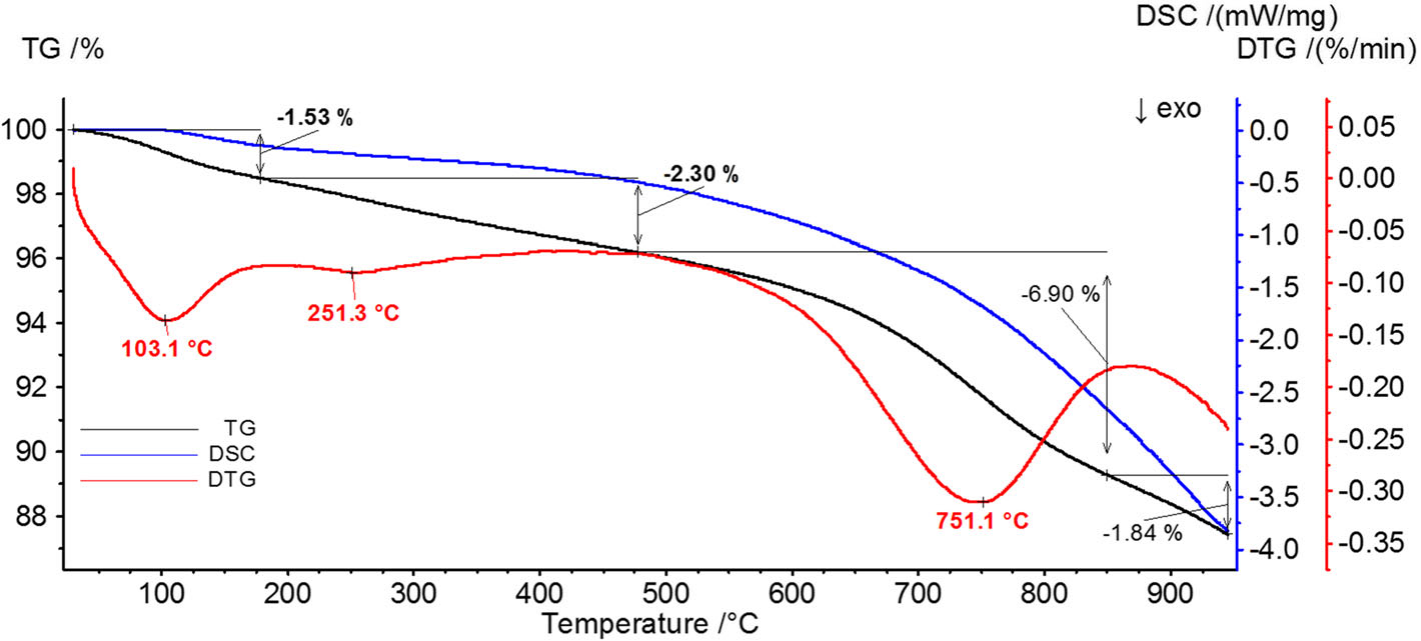
Thermogravimetric curves of activated carbon

Figure 2 shows the TG, DSC, and DTG curves of thermal decomposition of activated carbon. Four stages can be distinguished in the HPA heating process in the nitrogen atmosphere. The first one—in the range 30–180 °C with the minimum at 103.1 °C and the weight loss of 1.53%—is endothermic. It is associated mainly with removal of physically adsorbed water and the initial decomposition of the carbon carboxyl groups [33]. The second stage covers the temperature range 180–550 °C with a minimum of 251.3 °C on the DTG curve and the weight loss 2.3%. This phenomenon is associated with the decomposition of surface groups, such as carboxyl and sulfur-containing ones (e.g., sulfur-oxygen, sulfidic, benzene bound with the surface through disulfide bridges) [34]. In the temperature range of 550–850 °C (with the minimum 751.1 °C and the weight loss of 6.9%), there occurs further defragmentation of surface groups (lactone, quinone, and carboxylic anhydrides). The last temperature range, i.e., 850–945 °C, is associated with the weight loss of 1.48%. Apart from the processes’ characteristic of the previous stage, at a temperature above 800 °C, decomposition of strongly bound surface groups (such as carbonyl, pyrone, and ether) and surface oxidation of carbon can occur.

**Fig. 3:**
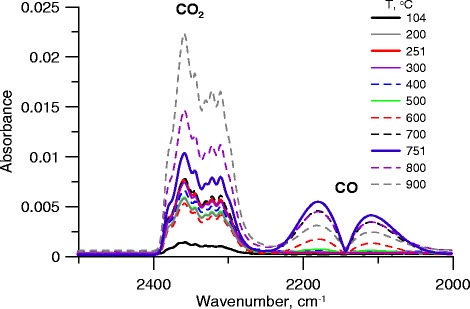
FTIR spectra of activated carbon. *Bold curves* are associated with the maxima on the DTG curve

Processes occurring in the temperature range of 550–950 °C were confirmed by the FTIR analysis. The FTIR spectra in the range characteristic of CO_2_ and CO (wavenumber 2000–3000 cm^−1^) are presented in Fig. 3. They indicate that above 600 °C, CO peak intensity increases, which proves the decomposition of quinone, carbonyl, pyrone, and ether groups of activated carbon [34].

**Fig. 4:**
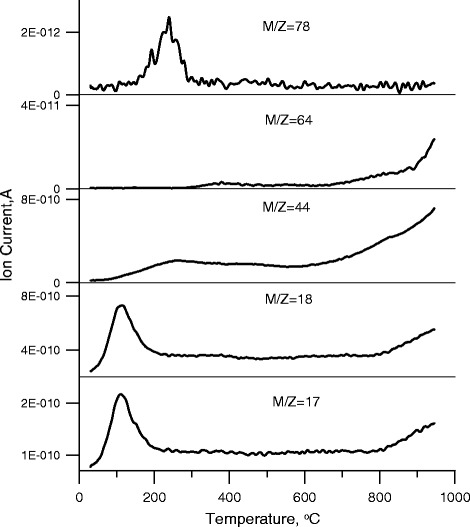
MS profiles of main products of activated carbon degradation

Figure 4 presents the MS profiles of main gaseous products of activated carbon decomposition: OH (m/z = 17), H_2_O (m/z = 18), CO_2_ (m/z = 44), SO_2_ (m/z = 64), and C_6_H_6_ (m/z = 78). In the low temperature range, i.e., 30–180 °C, there are two peaks with the maximum at 100 °C, characteristic of H_2_O (m/z = 18)—the removal of physically bound water and OH (m/z = 17)—removal of surface hydroxyl groups. In the 100–500 °C temperature range, there is a peak for CO_2_ (m/z = 44) with the maximum at 266 °C. The distinct peak for C_6_H_6_ (m/z = 78) is in the temperature range 100–300 °C with the maximum at 240 °C. In the temperature range 300–500 °C, there is a slight peak with the maximum at 378 °C and further intense increase at high temperatures above 700 °C for SO_2_ (m/z = 64). The peak corresponding to m/z = 44 is associated with decomposition of surface carboxyl groups [35]. The peak related to m/z = 78 corresponds to breaking off of the benzene rings (e.g., those associated with the activated carbon surface through the disulfide bridges). The decomposition of various types of surface groups containing sulfur is proved by the peak relating to m/z = 64.

### PAA Adsorption on the HPA Surface

**Fig. 5:**
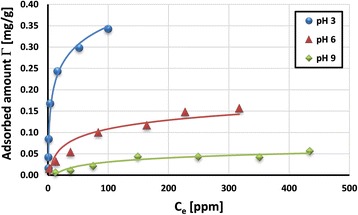
Adsorption isotherms of PAA 2000 Da on the HPA surface at various values of solution pH

**Fig. 6:**
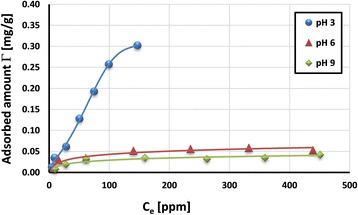
Adsorption isotherms of PAA 240,000 Da on the HPA surface at various values of solution pH

The adsorption isotherms of poly(acrylic acid) on the activated carbon surface are presented in Figs. 5 and 6. They were obtained at three solution pH values (3, 6, and 9). As can be seen, the higher the solution pH value is, the greater the adsorbed amount of PAA is. This is the result of changes in PAA carboxyl groups’ dissociation, as well as those of the sign and density of HPA surface charge.

The electrostatic attraction between the adsorbent and adsorbate occurs at pH 3. The dissociation of PAA functional groups at pH 3 is minimal, and for this reason, polymeric macromolecules assume a more coiled conformation. On the other hand, the surface of activated carbon is positively charged (point of zero charge of HPA is 7). Under such conditions, the structure of polymeric adsorption layer is characterized by considerable packing and as a result, the highest PAA adsorption level on the activated carbon surface is obtained.

The occurrence of favorable electrostatic forces does not exclude formation of hydrogen bridges between the adsorbent and adsorbate, as well as hydrophobic interactions. Dissociated and undissociated poly(acrylic acid) carboxyl groups can participate in the hydrogen bond formation with the activated carbon surface groups (especially those containing sulfur atoms). On the other hand, hydrophobic interactions can appear between the minimally dissociated polymer and the benzene rings (connected with the surface through disulfide bridges).

Additionally, PAA macromolecules with lower molecular weight (i.e., 2000 Da) can adsorb inside the solid pores (of mean diameter 2.26 nm) [36]. The evidence of this is slightly higher adsorption at pH 3 of PAA 2000 Da coils in comparison to the PAA 240,000 Da chains (Fig. 8). In the case of other metal oxides (i.e., zirconia), the thickness of PAA 2000 adsorption layer does not exceed 1.2 nm and hydrodynamic radius of polymeric coil in the solution was equal to 1.5 nm [37].

**Fig. 7:**
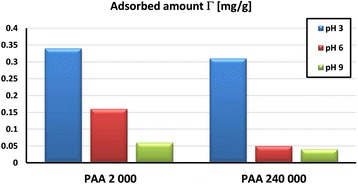
Comparison of adsorbed amount of PAA on the HPA surface at various values of solution pH

Significantly lower adsorbed amounts of poly(acrylic acid) are observed at pH 6 and 9. Despite the fact that at pH 6, there are still attractive interactions between PAA and the activated carbon surface, the conformation of polymeric chains changes significantly. Under such conditions, practically, total dissociation of PAA carboxyl groups occurs and polymeric chains assume a considerably extended conformation (in comparison to those at pH 3). Such conformation of adsorbed macromolecules consumes many active sites on the HPA surface, occupying a larger surface area. They block activated carbon surface groups, making them inaccessible to other adsorbing chains. Additionally, at pH 9, the electrostatic repulsion between the negatively charged adsorbent and totally dissociated PAA chains takes place. As a consequence, the significant decrease of PAA adsorption on the HPA surface is observed at pH 6 and 9. Considering the effect of polymer molecular weight, similar tendency to that at pH 3 was observed at pH 6 and 9 (Fig. 7). The PAA with lower molecular weight shows a greater adsorption level in comparison to the PAA with higher molecular weight. Although both polymers are characterized by extended conformation, the liner dimensions of PAA 2000 Da are considerably smaller than those of PAA 240,000 Da [26]. Thus, there is greater probability of penetration of solid pores (even partial) by polymeric chains with small size.

### Electrokinetic Properties of HPA Particles in the PAA Presence

**Fig. 8:**
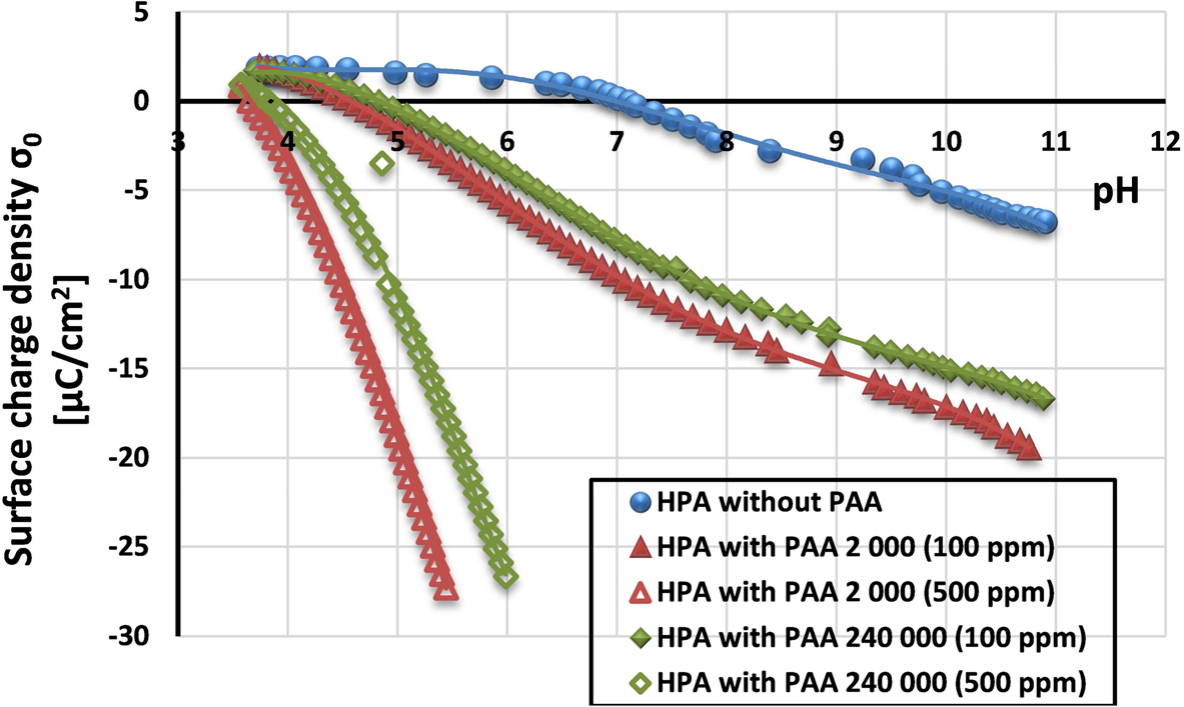
Surface charge density of activated carbon without and with poly(acrylic acid)—effect of polymer molecular weight and concentration

As can be seen in Fig. 8, the surface charge density (σ_0_) of HPA in the PAA presence decreases significantly (in comparison to the system without PAA). Moreover, the shift of pH_pzc_ to the lower pH values is observed (from pH 7 for HPA without PAA to pH 3.6 for HPA with PAA 2000 Da (500 ppm)). Additionally, reduction of σ_0_ value of activated carbon is greater in the case of lower molecular weight of PAA.

The main reason for such behavior of the examined systems is the presence of negatively charged carboxyl groups among the adsorbed polymer chains in the surface layer of the solution. Polymeric segments (containing –COOH groups) connected directly with the HPA surface groups (located in the train structures of adsorbed chains) cause an increase in the σ_0_ value. On the other hand, polymeric segments located in the tail and loop structures of adsorbed macromolecules contribute to the σ_0_ value decrease. Thus, the value of σ_0_ obtained experimentally is the sum of these two effects. The obtained results indicate that the effect of the presence of dissociated carboxyl groups belonging to the segments located in the tail and loop structures of the adsorbed PAA chains must be dominant in the examined systems in the whole range of studied pH (i.e., 3.6–11, Fig. 8).

What is more, flatter conformation of PAA with lower molecular weight makes their chains form shorter tail and loop structures. For this reason, the presence of a larger number of PAA dissociated carboxyl groups at the solid-liquid interface is responsible for more significant reduction of HPA surface charge density (in comparison to the system containing PAA 240,000 Da).

For both molecular weights of poly(acrylic acid), the increase in polymer concentration results in the decrease of the HPA surface charge density. This is caused by the increase in packing of polymeric adsorption layer which contains a larger number of negatively charged carboxyl groups.

**Fig. 9:**
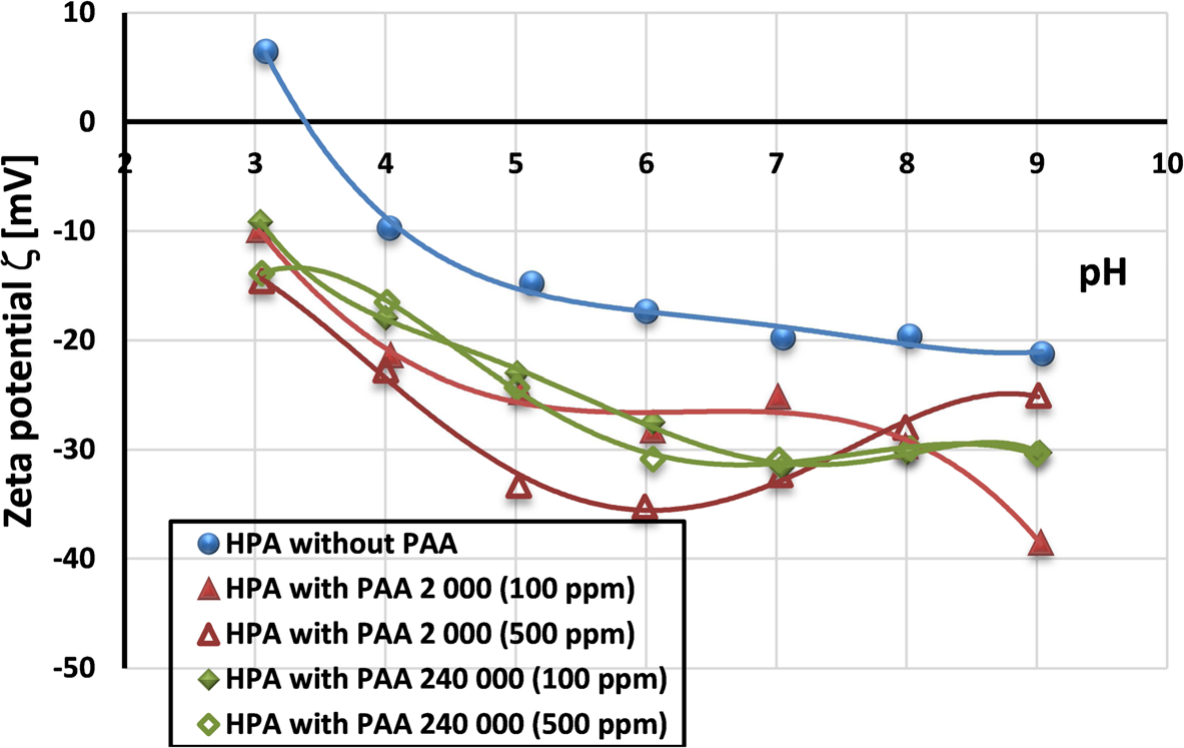
Zeta potential of HPA particles without and with poly(acrylic acid)—effect of polymer molecular weight and concentration

Analysis of the zeta potential measurement results (Fig. 9) leads to the conclusion that adsorption of poly(acrylic acid) on the HPA particles’ surface causes reduction of electrokinetic potential value. What is more, the isoelectric point of the solid without the polymer is located at pH 3.4. Such big difference between pH_pzc_ and pH_iep_ values of activated carbon may be a result of overlapping of the electrical double layers formed on the mesopore walls [38, 39].

According to M’Pandou and Siffert [40], the main reason for the zeta potential lowering in the anionic polymer presence is the occurrence of charged groups (negative carboxyl ones) of the polymer macromolecules in the area of slipping plane. The shift of slipping plane due to the presence of polymeric adsorption layer is the second reason for the zeta potential decrease. This effect is dominant under the conditions in which polymer macromolecules assume more extended conformations on the solid surface (i.e., at higher PAA concentrations). The third phenomenon is associated with the blockade of active sites on the solid surface by the adsorbed macromolecules. Such behavior is caused by flat conformation of polymeric chains on the activated carbon surface. Thus, the experimentally obtained value of electrokinetic potential of solid particles covered with polymer depends on the contributions of these effects.

## Conclusions

The nanostructure of poly(acrylic acid) adsorption layer formed on the activated carbon obtained by physical activation of residue after supercritical extraction of hops (HPA) was characterized. It was demonstrated that PAA with a lower molecular weight (2000 Da) exhibits the highest adsorption at the solid-liquid interface at pH 3. Under such conditions, the most coiled structure (slight dissociation of polymer carboxyl groups) is assumed by PAA macromolecules. The electrostatic attraction between the positively charged solid surface and the polymeric coils leads to the formation of an adsorption layer composed of densely packed macromolecules. Moreover, at pH 3 PAA, macromolecules with lower molecular weight can penetrate solid pores of the mean diameter 2.26 nm. Besides electrostatic forces, the hydrogen bridges’ formation and hydrophobic interactions can participate in the polymer adsorption process on the surface of activated carbon.

Poly(acrylic acid) adsorption on the HPA surface causes decrease in both surface charge density and zeta potential of the solid particles (in comparison to the system without polymer). The main reason for such behavior is the presence of negatively charged polymeric segments (belonging to adsorbed macromolecules) both in the surface layer of the solution (leading to the decrease of solid surface charge density) and in the area of slipping plane (resulting in reduction of zeta potential).
